# A Patent and Pattern Mother

**Published:** 1874-03

**Authors:** 


### A PATENT AND PATTERN MOTHER.

A physician of superior abilities in a neighboring state had a daughter of sweet seventeen, whose health was not as good as was desired, and not wishing to give her medicine, he made arrangements for having her travel leisurely through New England for several months, as the most likely means of her restoration. At the same time her mother obtained a supply of various patent medicines, and placed them in the travelling trunk, *with private instructions* to take them while travelling. The daughter to accommodate herself and her father, took the journey, and in obedience to the mother, took the physic, and as a matter of course, is—still an invalid. The father remains in ignorance of the cause of the failure of beneficial results from his prescription.

LESSON.—*The child who privately does a thing for one parent, known to be contrary to the wishes of the other, and the parent who can counsel it, are equally guilty of a violence against domestic rule, which will not cease to bear the pernicious fruits of deception and discord, to the latest hour of their lives.*

